# Intranasal delivery of a Fas-blocking peptide attenuates Fas-mediated apoptosis in brain ischemia

**DOI:** 10.1038/s41598-018-33296-z

**Published:** 2018-10-09

**Authors:** Irfan Ullah, Kunho Chung, Jungju Oh, Jagadish Beloor, Sumin Bae, Sangah Clara Lee, Minhyung Lee, Priti Kumar, Sang-Kyung Lee

**Affiliations:** 1 Department of Bioengineering and Institute of Nanoscience and Technology, Hanyang University, Seoul, Korea; 2 Department of Internal Medicine, Section of Infectious Diseases, Yale University School of Medicine, New Haven, CT USA; 3 Department of Behavioral and Social Sciences, Brown University, Providence, RI USA

## Abstract

Ischemic stroke-induced neuronal cell death results in the permanent disabling of brain function. Apoptotic mechanisms are thought to play a prominent role in neuronal injury and ample evidence implicates Fas signaling in mediating cell death. In this study, we describe the neuroprotective effects of a Fas-blocking peptide (FBP) that by obstructing Fas signaling in cerebral ischemia inhibits apoptosis. Using an intranasal administration route in a rat model of focal cerebral ischemia, we demonstrate that nose-to-brain delivery of FBP after middle cerebral artery occlusion (MCAO) surgery results in the delivery and retention of FBP in Fas-expressing ischemic areas of the brain. A single intranasal administration of 2 mg/kg FBP resulted in significantly reduced neuronal cell death by inhibiting Fas-mediated apoptosis leading to decreased infarct volumes, reduced neurologic deficit scores and recovery from cerebral ischemia. Intranasally delivered FBP might be a promising strategy for the treatment of cerebral ischemic stroke.

## Introduction

Ischemic stroke is the third major cause of death and permanent disability worldwide^1^. Globally, ischemic stroke is estimated to be responsible for 9.5% of all deaths and 15 million people suffer stroke each year according to world health organization^2,3^. Of these, 5 million die and another 5 million are permanently disabled. In about 85% of the patients, ischemic stroke occurs from occlusion of a major cerebral artery, commonly the middle cerebral artery (MCA), by thrombus or embolism. The effects of ischemia are fairly rapid because the brain does not store glucose, the chief energy substrate, and is incapable of anaerobic metabolism^4^. Despite greater than a decade of work, facilitation of functional recovery after stroke is limited. The current therapeutic approach for ischemic stroke is the use of tissue plasminogen activator (tPA), approved by FDA, however a favorable outcome is incumbent upon early treatment (<4 hours of ischemic onset) and less than 5% of qualifying patients actually receive TPA^5^.

There is ample evidence to suggest that apoptosis, in addition to coagulation necrosis, contributes to the neuronal cell death that occurs after brain ischemia^6^. Ischemic stroke is recognized to trigger two main pathways of apoptosis- the intrinsic pathway originating from mitochondrial release of cytochrome *c* and an extrinsic pathway that occurs by signaling through cell death receptors that belong to the tumor necrosis factor receptor (TNFR) superfamily such as Fas (Apo-1, CD95), tumor necrosis factor (TNF)-related apoptosis inducing ligand-R1 (TRAIL-R1), TRAIL-R2 and TNFRp55^7^. Among TNFRs, the Fas ligand/receptor signaling pathway has been implicated as critical in triggering apoptotic signals in acute ischemia. A number of studies demonstrate the up-regulation of both Fas and FasL in the ischemic penumbra in animal models of focal ischemia^8–12^. FasL mutant mice (gld) are strongly protected from cerebral ischemic injury, and FasL deficiency does not appear to affect brain development or anatomy in these mice^13^. Further, rats lacking functional Fas (lpr), the receptor for FasL exhibit a profound reduction in infarct size and improved survival after ischemic stroke injury^14^. Further treatment with anti-Fas antibody led to reduction in infarct volume in a mouse model of ischemic injury model^13^. These findings present strong evidence that Fas ligand/receptor pathway promotes cell death following brain ischemia and inhibition of Fas ligand/receptor interaction may provide significant neuroprotection affording a new treatment modality in ischemic stroke injury.

Delivery of drugs through the systemic route to the brain is restricted due to the presence of blood brain barrier (BBB), which is composed of specialized endothelial cells that are selective in permitting diffusion into the central nervous system (CNS). Several approaches have been investigated to enable drug delivery through the BBB^15,16^. A lot of effort has been invested in the development of intravenous (IV) transport systems such as the glucose transporter^17–19^ or ones based on receptor-mediated delivery such as transferrin, insulin, or acetylcholine that may enable transcytosis of therapeutic molecules attached to ligands after binding the receptor on the BBB^15,20^. However these approaches are limited to certain types of therapeutic molecules and are also hampered by issues of effective drug delivery due to rapid plasma clearance, slow diffusion across the BBB and reverse transcytosis from the brain to blood^16,20,21^.

Recently, the intranasal (IN) route, which bypasses the BBB, has garnered a lot of interest as a means to access the CNS^22^. IN delivery of drugs to brain is a complex mechanism involving the olfactory, trigeminal nerves and cerebrospinal fluid (CSF) as conduits for transporting molecules from nasal cavity to the CNS by bypassing the BBB^23^. The extensive distribution of olfactory and trigeminal nerves in the nasal epithelium allows uptake and direct transport to various brain regions^23^. Animal studies have revealed that a wide variety of therapeutic molecules such as nucleic acids, peptides and drugs formulated in nanoparticles or in free-form can be successfully delivered to the brain by IN administration in various animal models of disease such as ischemic stroke, glioblastoma, and neuroinflammation^24–26^. Furthermore, the clinical relevance of this method is high given the non-invasive route of delivery and absence of side effects that are associated with systemic administration^27,28^. Additionally, this method provides the advantage of multiple dosing with a reduced risk of immunogenicity, cost-effectiveness and even the possibility of use without clinical supervision^29^.

In this study we investigated if blockade of Fas-FasL interaction by IN delivery of a Fas-blocking peptide (FBP)^30^ would prove therapeutic rescuing from neuronal cell death in ischemic stroke. Our results demonstrate that FBP, upon intranasal administration, specifically binds Fas-expressing apoptotic regions in the brain in a rat ischemia model and effectively blocks Fas-mediated apoptosis resulting in recovery from ischemic stroke.

## Results

### Fas Blocking Peptide blocks Fas-mediated apoptosis

**Figure 1:**
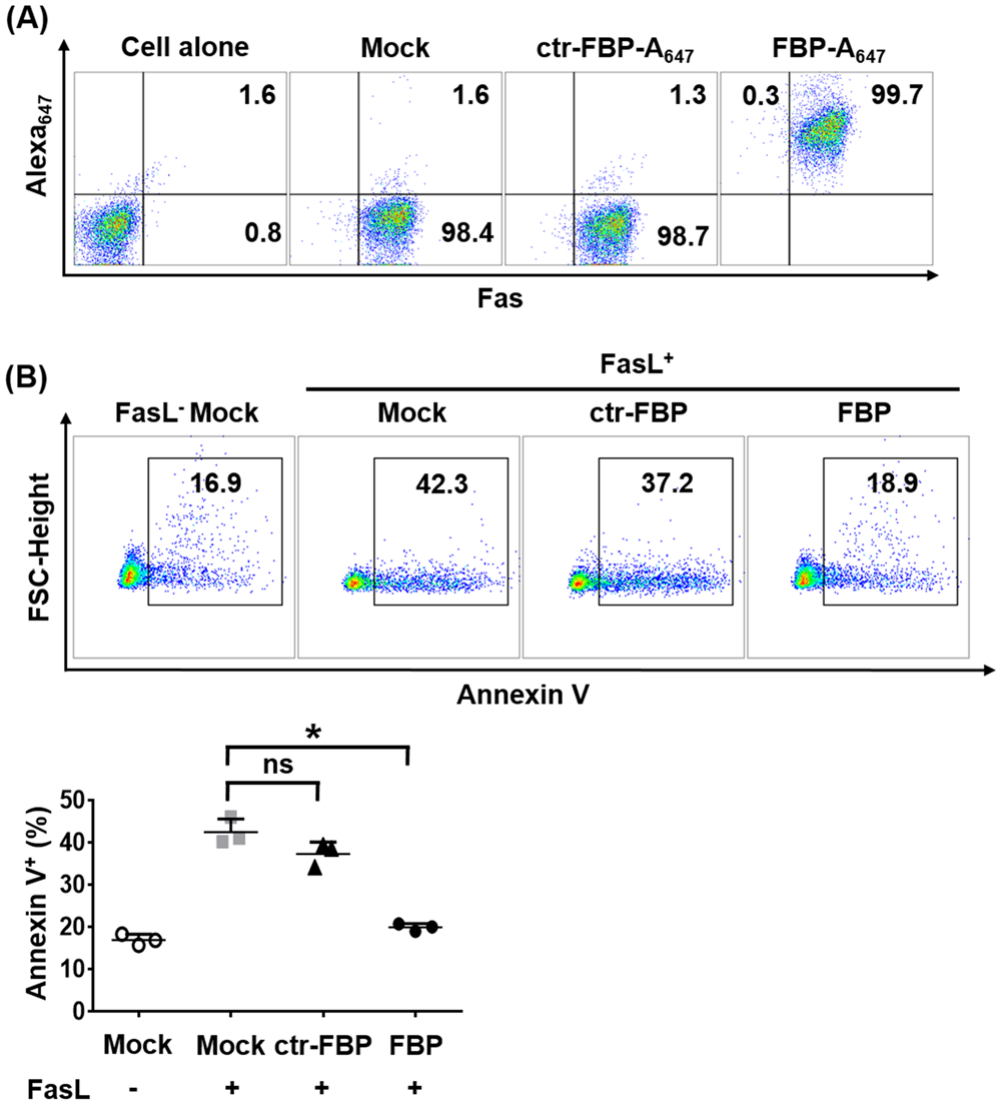
FBP binds Fas-expressing Jurkat cells and inhibits FasL-induced apoptosis. (**A**) Flow cytometric analysis of Fas-expressing Jurkat cells bound to a rabbit anti-rat Fas antibody and/or indicated A_647_-peptides. Cells untreated with Fas antibody are indicated as ‘cells alone’ and the ‘cells stained anti-Fas antibody’. A_647_-labelled control peptide or A_647_-labelled FBP peptide are indicated as ‘Mock, ctr-FBP-A_647_ and FBP-A_647_’ respectively. (**B**) Anti-apoptotic effect of Fas-blocking peptide. Cells were exposed to FasL in the presence or absence of FBP. Representative dot plots for one experiment are shown and cells were scored as positive for Annexin V (black box). Cumulative data are in scatter plots depicting the % annexin V positive cells (lower panel). The data represents mean ± SD. *P < *0.05*; ns- not significant; Mann-Whitney U test. FasL- Fas ligand, ctr-FBP- control peptide, FBP- Fas blocking peptide.

A cyclic Fas-blocking peptide (FBP) was demonstrated in a previous study to disrupt Fas-FasL interactions and inhibit Fas-mediated apoptosis^30^. Our studies utilize a linear form of this peptide rather than the previous cyclic form^30^ as the linear form of peptide was found to be equally potent in activity in *in vitro* studies (Supplementary Fig. S1A,B). We additionally confirmed by the Ellman’s assay that >98% of the peptide was in a free linear form and storage in frozen conditions does not result in oxidation prior to use in experiments (Supplementary Fig. S1C). To test the functional activity of linear FBP, we first analyzed its binding and anti-apoptotic properties in Jurkat cells that constitutively express Fas^30^. FBP (A_647_-labeled) effectively bound Fas-expressing Jurkat cells and protected these cells from FasL induced apoptosis as determined by reduced staining with Annexin V (Fig. 1A,B).

### FBP blocks apoptosis in the Neuro2a cell model of ischemia

**Figure 2:**
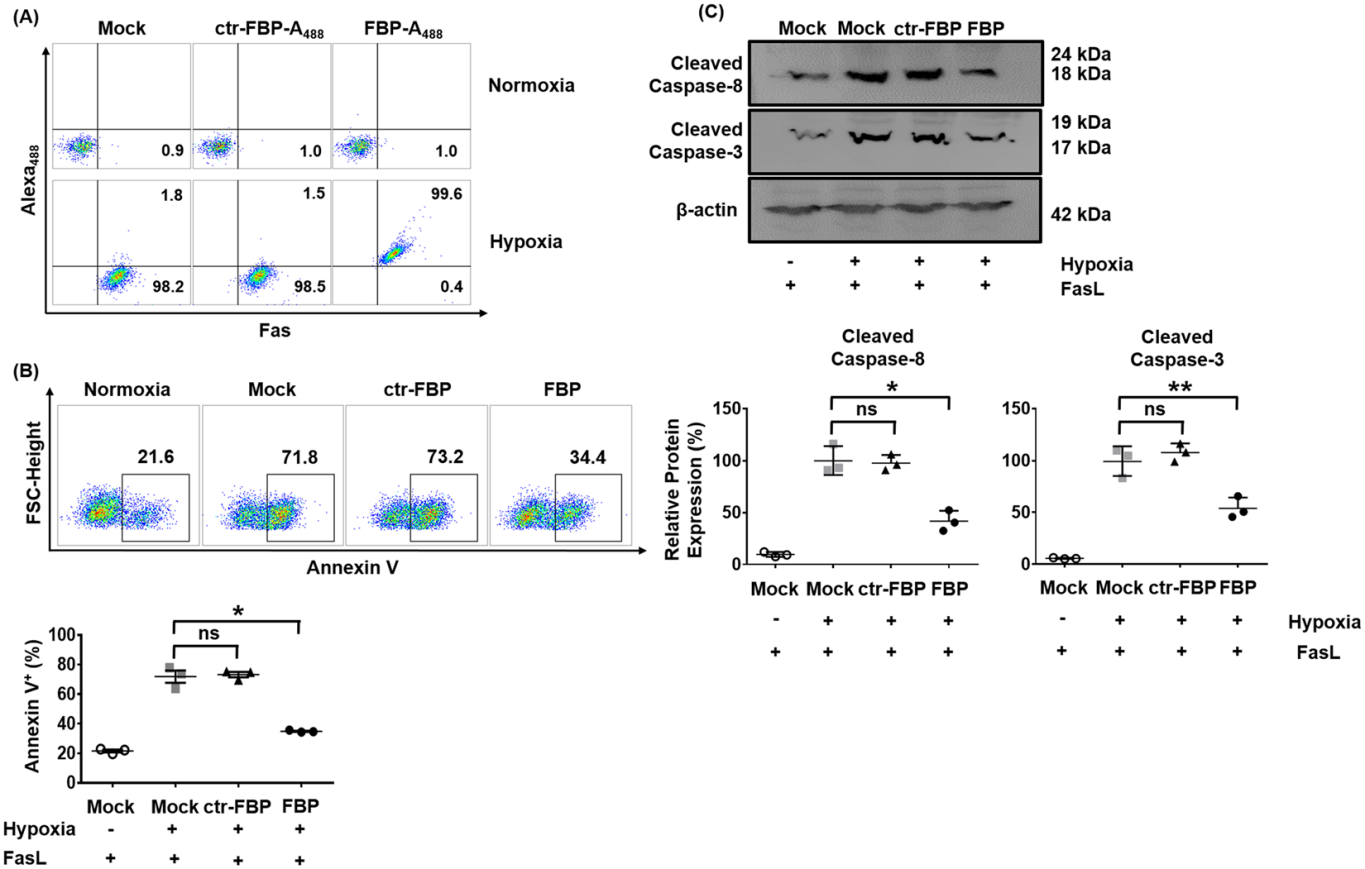
FBP reduces hypoxia-induced apoptosis of Neuro2a cells. (**A**) Dot plots representing one of three experiments depicting binding of FBP to Fas-expressing Neuro2a cells. Normoxia refers to Neuro2a cells maintained under normal culture conditions and hypoxia to Neuro2a cells maintained for 24 h in OGD media under hypoxic conditions followed by a 4 h re-oxygenation period. Cells stained with anti-Fas antibody, A_488_-labeled control peptide and A_488_-labeled FBP peptide prior to flow cytometric analysis are indicated as Mock, ctr-FBP- A_488_ and FBP- A_488_ respectively. (**B**) Flow cytometric analysis for apoptosis in Neuro2a cells. Representative dot plots for one experiment are shown and cells were scored as positive for Annexin V (black box). Cumulative data are in scatter plots depicting the % annexin V positive cells (lower panel). The data represents mean ± SD. *P < *0.05*; ns- not significant; Mann-Whitney U test. (**C**) Western blots for cleaved caspase-3 and caspase-8 proteins. The numbers represent apparent molecular weights of the protein bands. Full non-cropped blots are presented in Supplementary Fig. S4. Cumulative data is shown below depicting levels of cleaved caspase 8 or 3 relative to that in mock-treated hypoxic cells. Protein expression was obtained by normalizing band intensities to that of β-actin measured in arbitrary pixel values using the ImageJ software. The data represents mean ± SD (N = 3 individual values from separate experiments). *P < 0.05, **P < *0.01*; ns- not significant; Mann-Whitney U test. FBP- Fas blocking peptide, ctr-FBP- control peptide, FBP-A_488_- Alexa_488_ labeled FBP.

We next investigated the effects of FBP-mediated Fas-blockade in a commonly used *in vitro* model for brain ischemia comprising mouse neuroblastoma, Neuro2a cells cultured under conditions of oxygen and glucose deprivation (OGD)^13^. A_488_-labeled FBP, and not the control peptide, ctr-FBP, bound to hypoxic cells expressing Fas but not to cells maintained under normoxic conditions (Fig. 2A). Confocal imaging data also revealed that A_647_-labeled FBP bound to only Fas-expressing hypoxic Neuro2a cells but not ctr-FBP (Supplementary Fig. S2). We next evaluated the extended effects of FBP on Neuro2a cells that displayed elevated levels of Annexin V under hypoxic conditions. Treatment of Neuro2a cells with FBP 4 h post hypoxia and re-oxygenation dose-dependently reduced cell death after exposure to FasL with annexin V positivity decreasing by 47% at higher concentrations of upto 1 mM (Fig. 2B, Supplementary Fig. S3). Cleavage of caspase-3 is an important trigger in hypoxia-induced apoptosis^31,32^ and hypoxic Neuro2a cells had large increases in the levels of cleaved caspase 3 as well as caspase 8 (Fig. 2C, Supplementary Fig. S4). FBP treatment (1 mM) attenuated the levels of cleaved caspase 3 and 8 as shown in Fig. 2C. The ctr-FBP did not reduce levels of these cleaved enzymes in the cells. Collectively, our data suggest that FBP binds Fas-expressing cells and rescues them from Fas-mediated apoptosis.

### Intranasal delivery of Fas-blocking peptide attenuates apoptosis

**Figure 3:**
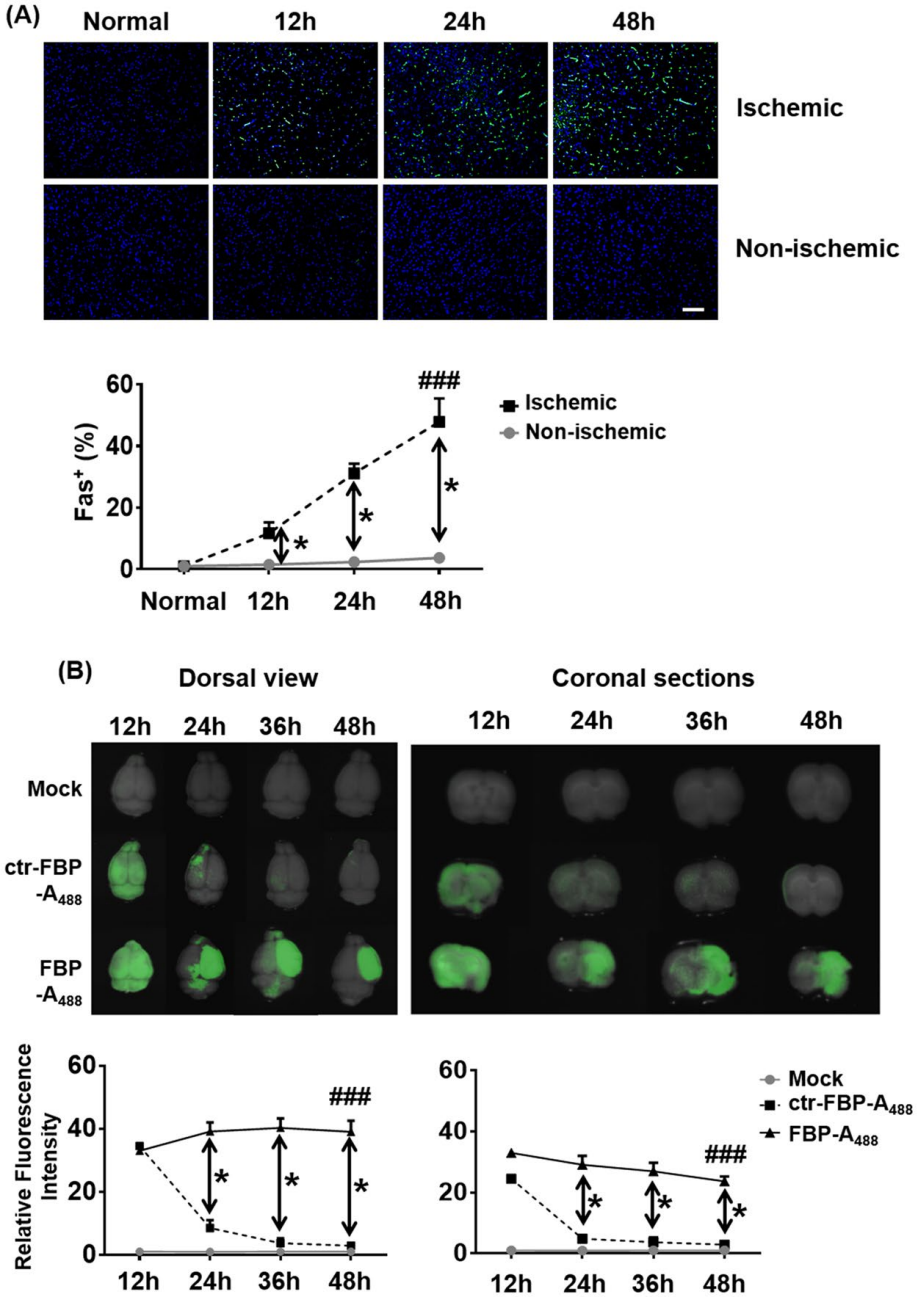
IN delivered FBP localizes to Fas-expressing brain regions in the MCAO rat model of focal ischemia. (**A**) Immunohistochemistry for Fas expression in ischemic rat brain after MCAO. Representative images of deparaffinized coronal brain sections from normal or ischemic rats stained with anti-Fas antibody at the designated times post-MCAO are shown. Representative images (upper panel) and cumulative data (lower panel) depicting fluorescence intensities measured in arbitrary pixel values relative to that at the 12 h ‘normal’ rats. Scale bar = 50 µm. The data represent mean ± SD (N = 6 per group). *P < *0.05* at individual time-points, Mann-Whitney U test, ^*###*^*P* < *0.001*; Cumulative data, Two-way ANOVA. (**B**) Brain biodistribution of intranasally inoculated FBP in ischemic rats. Brain from MCAO rats that were treated with PBS, ctr-FBP or FBP were visualized dorsally (top view) and as coronal sections (coronal view) for the presence of fluorescent label representing FBP-A_488_ or ctr-FBP-A_488_ at the indicated times post-inoculation. Representative images (upper panel) and cumulative data (lower panel) depicting fluorescence intensities measured in arbitrary pixel values relative to and normalized based on fluorescence intensities in normal group. The data represent mean ± SD (N = 6 per group). *P < *0.05* at individual time-points, Mann-Whitney U test, ^*###*^*P* < *0.001*, Cumulative data, Two-way ANOVA; ns- not-significant.

Clinical data suggest that Fas-mediated apoptosis may be the dominant cause for cell death in human ischemic stroke as apoptotic cells appear to line the peri-infarct area in the human brain after ischemic stroke and a linear correlation exists between the neuronal expression of Fas- FasL and cell death^33^. Further, induction of several intermediary effectors of apoptotic cell death described in animal experiments (Fas, FasL, Caspase 3, ACA-3, PARP-1) were also observed in human ischemic stroke^33,34^. We sought to investigate the effects of FBP in a rat model of focal cerebral ischemia after middle cerebral artery occlusion (MCAO). To confirm onset of ischemic stroke, cerebral blood flow was measured during ligation, occlusion and reperfusion and a dramatic reduction of blood flow (~90%) was recorded upon occlusion of the MCA (Supplementary Fig. S5A). Ischemic stroke condition elevates the Fas mRNA and protein expression in rat brains after few hours of MCAO^8,10^. Upon right MCAO, which induces ischemia in the right side of the brain, by post-euthanasia immunohistochemistry of brain tissue, we found a time-dependent increase in Fas expression selectively in right hemisphere (Fig. 3A). Compared to the right hemisphere (ischemic), negligible Fas expression was observed in left hemisphere (non-ischemic) after each time point as only the right hemisphere becomes apoptotic after right MCAO procedure as expected (Fig. 3A). To target Fas *in vivo* we utilized the IN route to deliver FBP directly to the brain using a pressurized olfactory device (POD)^35^. Intranasal inoculation of A_488_-labeled FBP resulted in brain localization of the fluorescence labeled peptide in normal as well as MCAO-induced rats at 12 h (Fig. 3B and Supplementary Fig. S5B). However, by about 24 h post inoculation, A_488_-labeled FBP persisted only in the brains of MCAO-treated rats and that too in the right. These data indicate that FBP retention in the ischemic areas of that MCAO rat brain maybe due to binding of FBP to Fas-expressing neuronal cells.

**Figure 4:**
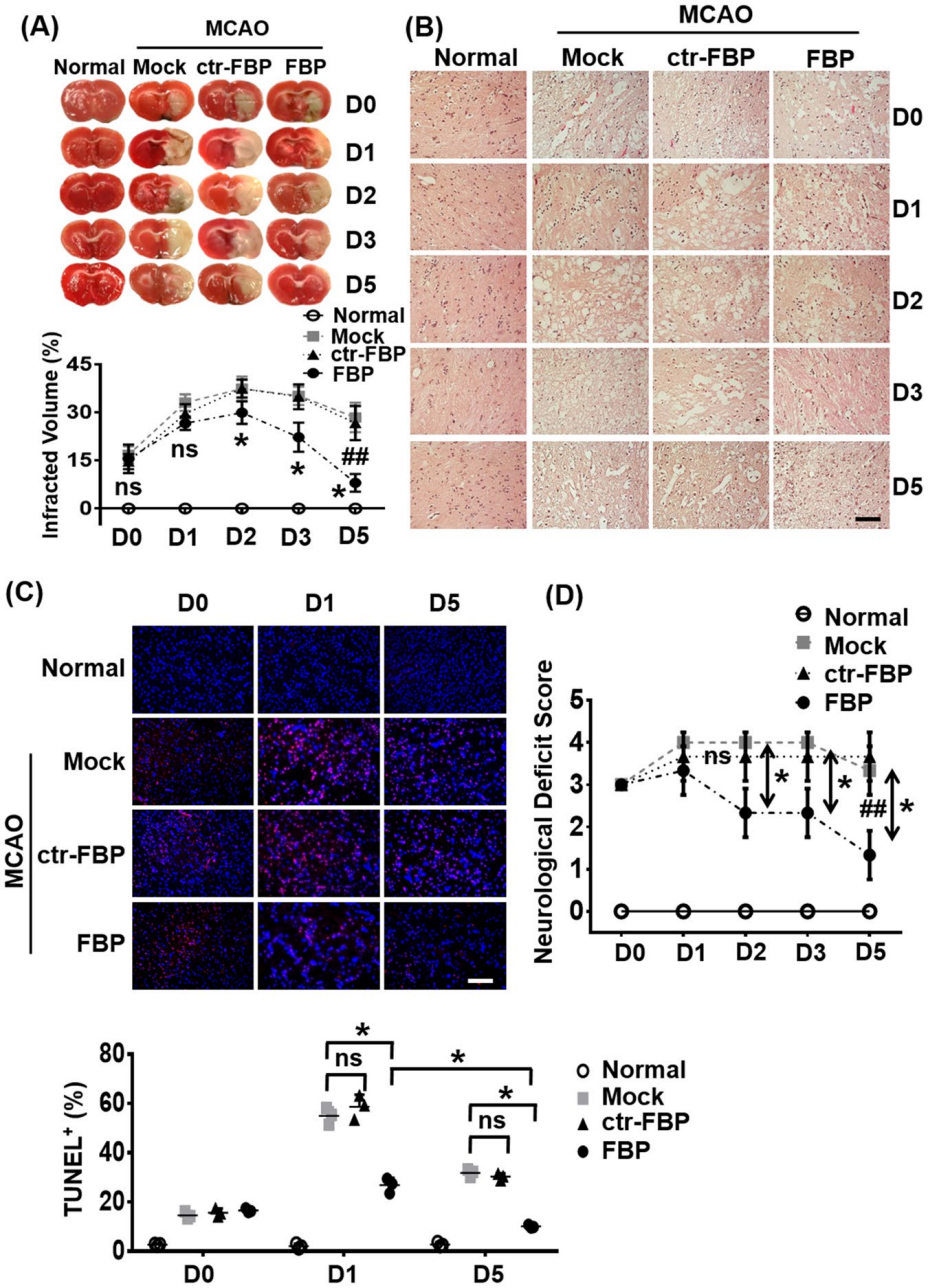
IN delivered FBP blocks apoptosis in rats with focal brain ischemia. (**A**) Brain slices stained with TTC after ischemia. Quantitative analysis is shown below. The data represent mean ± SD (N = 6 per group). *P < *0.05* at each individual time-point, Mann-Whitney U test, ^*##*^*P* < *0.01*; Cumulative data, Two-way ANOVA; ns- not-significant. (**B**) Representative images of hematoxylin and eosin (H&E) brain sections from normal or MCAO subjected animals after treatment. Scale bar = 100 µm (**C**) Representative images of TUNEL-stained brain sections (upper panel) showing TUNEL positive dead cells (red) and DAPI stained nuclei (blue) in normal or ischemic region of MCAO induced rats. Scale bar = 100 µm. Quantitative analysis of (**C**) is shown below. The data represent mean ± SD (N = 6 per group). *P < *0.05* at individual time-points, Mann-Whitney U test, ^*##*^*P* < *0.01*; Cumulative data, Two-way ANOVA; ns- not-significant. (**D**) Neurological deficit scores at indicated days after ischemia. Mock- PBS treatment. The data represents mean ± SD (N = 6 per group). *P < *0.05* at across groups at each time point, Kruskal-Wallis test, ^*##*^*P* < *0.01*, Cumulative data, Two-way ANOVA; ns- not-significant.

Ischemic stroke condition in rat models causes brain damage by inducing cell death predominantly through apoptosis^8,33^. TTC-stained coronal slices of brain tissue from MCAO rats analyzed day 1–5 post-MCAO on a daily basis demonstrated a significant infarct size as early as 12 h post-MCAO (day 0, Fig. 4A, upper panel) evidenced by a white TTC-negative area. The infarct size increased prominently to almost the entire right hemisphere of the brain by 24 h post-MCAO (day 1). The brain slices from rats that did not undergo MCAO (normal) and contralateral brain regions from MCAO rats did not show signs of infarction (Fig. 4A). To evaluate effects of FBP on brain infarction, animals were administered 2 mg/kg IN at 12 h post-MCAO. In mock (PBS)-treated groups, we found severe cerebral infarction ~32% after day 1 post-MCAO and % of infarction volume was 37%, 35% and 28% on day 2, 3 and 5 respectively (Fig. 4A, lower panel). Compared to mock (PBS) or ctr-FBP treatment, FBP treatment significantly reduced the size of the infracted area as early as day 1 post-MCAO and an almost complete recovery from infarction occurred by day 5 post-MCAO. The infarction recovery in FBP inoculated groups was 10%, 20%, 37% and 75% after day 1, 2, 3 and 5 respectively. H&E staining of brain sections also revealed significant damage to the tissue morphology in the right hemisphere of the brain as early as 12 h post-MCAO in mock and ctr-FBP-treated rats which progressively increased over time by day 5 post-MCAO (Fig. 4B). In contrast, tissue damage was reduced at day 1 and highly attenuated at day 5 post-MCAO in FBP inoculated groups. The levels of TUNEL positive cells, which approximated 15% in all groups at 12 h post-MCAO, increased to 54% and 32% in mock- and 56% and 37% in ctr-FBP-treated groups at days 1 and 5 post-MCAO respectively. FBP treatment significantly reduced the number of apoptotic cells to 26% and 9% at day 1 and 5 post-MCAO (Fig. 4C). Brains of non-ischemic rats did not show any morphological changes indicating that IN inoculation of peptide did not by itself affect the brain tissue (Supplementary Fig. S5C, upper panel). The absence of TUNEL positive cells in contralateral region of brain also revealed that apoptosis is specific to ischemic brain region (Supplementary Fig. S5C, lower panel).

Rats subjected to MCAO exhibit neuro-functional defects post-MCAO that can be graded on a clinical scale and assigned neurological deficit scores^36^. Our study showed a progressive debilitating effect on behavior as early as 12 h post-MCAO that worsened by day 1–5 in both mock- and ctr-FBP inoculated groups. All the animals displayed symptoms of circling to the paretic side when pulled by the tail (grade 3) within 12 h post-MCAO that progressively worsened and became spontaneous (grade 4) in day 1–3 post-MCAO (Fig. 4D). The neurological deficit scores improved in FBP treated groups compared to other ischemic groups (Fig. 4D). The neurological function in FBP treated groups were graded an average of 3.3, 2.3, 2.3 and 1.3 at day 1, 2, 3, 5 post-MCAO respectively that were 1.1, 1.5, 1.5, 2.8 fold lower that control groups at these time points respectively. Altogether, our results demonstrate that blocking the Fas signaling cascade using FBP not only reduces apoptosis but also improves recovery and reduces neurological deficits in rats subject to ischemic stroke.

## Discussion

Ischemic stroke injury triggers multiple signaling pathways and both necrosis and apoptosis contribute significantly to induce neuronal cell death. After focal cerebral ischemia, cells in the ischemic core undergo necrosis and cell death in the ischemic penumbra is by an active apoptotic process and thus a possible target for neuroprotective treatments^8,33^. Several molecules involved in the TNF/Fas family death receptor-mediated extrinsic pathway including Fas and FasL are increased, both at the transcriptional and protein levels, particularly in the ischemic penumbra, very early after focal cerebral ischemia and remain elevated or further increase in experimental animal models^8–10^. Studies on a unique set of human post-mortem brains from fatal ischemic stroke cases also provide evidence of the same pathways of cell death encouraging the treatment of human ischemic stroke with anti-apoptotics for a favorable outcome^33,34^. Accordingly, antibodies to FasL can prevent primary ischemic injury and when used in combination with anti-TNF antibodies, they also reduce secondary inflammatory injury in ischemic mice^13^. Pharmacological inhibition of the apoptosis effector enzyme caspase-8 with the inhibitor TRP801 also provides significant neuroprotection against hypoxia-induced brain injury in rats. Thus, blocking Fas ligand/receptor interaction may provide significant neuroprotection and our studies demonstrate the use of FBP in this regard as a new treatment modality for ischemic stroke injury.

Traditional approaches for drug delivery to the brain such as intracranial and stereotactic injections are impractical and invasive. In contrast, the IN administration route is an attractive non-invasive convenient option for targeted delivery to the CNS and limits the side effects associated with peripheral administration of drugs^37–39^. A variety of molecules have been demonstrated to reach the brain tissue when administered through IN route and some at concentrations effective to treat neurological disorders^37,38,40–43^. In the context of cerebral ischemia, IN treatment with pharmacological inhibitors of caspases^44,45^, proteins/hormones like glucocorticoids, mesenchymal stem cells and their secreted factors, IL-1 receptor antagonist^46–50^, peptides such as complement-derived peptide C3a, apelin 1, exendin 4^46,51,52^, to name a few have all been demonstrated to have beneficial effects in animal models.

More than 45 clinical trials are underway using the IN approach to treat brain-associated disorders based on pre-clinical evaluation in animal models (www.clinicaltrials.gov). However, the significant differences in the anatomy of the nasal cavity in humans when compared to rodents^39,53,54^ has limited the efficacy of the IN delivery approach in clinical trials^55,56^. The relative surface area of the nasal cavity in mice and rats is higher by a factor of 15 and 8 respectively compared to humans and the olfactory epithelium (expressed as percent of nasal cavity surface area) is also 6 times higher (Reviewed in^55^). However, it has been difficult to discern the effective IN drug dosing in humans based on these criteria. For example, ~2 U of insulin was sufficient to treat Alzheimer disease in rats, while in clinical testing, 10–20 U of insulin gave promising results (clinical trial ID: NCT01547169 for Phase II and NCT01767909 for Phase II/III)^57^. On the other hand, the GLP-1 peptide hormone was used at ~8–16 μg in animal studies^58^, while in a Phase III study in humans, ~3 mg was considered appropriate for weighting at 50 Kg (clinical trial ID: NCT01994746). A neuroprotective protein, ADNP, an eight amino acid long peptide has completed Phase ΙΙ clinical trials (clinical trial: NCT00505765) for IN treatment at 5 and 30 mg/day of patients with mild cognitive impairment, schizophrenia and Alzheimer disease based on experiments performed in mice using 0.5–2 µg/mouse/day)^59,60^. Thus, while the clinical relevance of this method is promising, it is difficult to predict the dosing for human studies with experimental therapeutics like the FBP. With newer approaches like mucosal flap reconstruction and pressurized olfactory devices for drug deposition at sites beyond the nasal valve where the absorptive mucosal epithelium is located^61,62^ nose-to-brain delivery may elicit promising results at a dosing that is more in concert with dosing in rodents.

Our study is the first demonstration that direct inhibition of Fas-FasL interactions with FBP can treat ischemic stroke after a single IN treatment highlighting the important role of the Fas-apoptotic pathway in ischemic injury. Furthermore, we find that IN delivery results in extensive permeation of the brain tissue with the FBP peptide and localization in the Fas-expressing ischemic core of MCAO rats. Interestingly, while this extent of distribution may be attributed to temporary disruption of the nasal epithelial barrier that can occur in an ischemic environment and the experimental conditions used, the non-targeting control peptide however was cleared within a few hours of treatment demonstrating the specificity of the approach. The clearance of non-binding peptide may have occurred through absorption into the cerebrospinal fluids^63–65^ or via the vascular pathway through perivascular spaces that act as lymphatic system for the brain^23^, which needs to be elucidated.

Collectively, our study demonstrates that the Fas pathway is a key trigger of neuronal cell death in the ischemic stroke model and blockade of Fas signaling by FBP through nose-to-brain delivery efficiently protects from ischemic brain damage.

## Conclusion

Our study demonstrates that intranasal administration of a Fas-blocking peptide to Fas-expressing ischemic regions in the brain results in selective delivery of FBP and effectively protects from ischemic stroke-induced neuronal cell death. Thus, our data provides a non-invasive treatment approach targeting the Fas pathway as a novel therapeutic for ischemic stroke.

## Materials and Methods

### Peptides

The peptides used in this study have been described previously and were custom synthesized from Peptron Co. (Daejeon, Korea)^30^. The peptide supplied in powder form was dissolved in PBS (pH 7.4) upon receipt and stored frozen at −70 °C for use in experiments. The sequences are depicted below;

Fas blocking peptide (FBP): YCDEHFCY

Control peptide (ctr-FBP): YCNSTVCY

The peptides were used in the linear form and not after cyclization as reported previously^30^ as we found no differences in activity *in vitro*. The linear structure of peptide was confirmed by the Ellman’s assay (Thermo fisher scientific, Carlsbad, CA) which uses 100 mM DTNB [(5, 5′-dithiobis-(2-nitrobenzoic acid)] according to the manufacturer’s instruction.

### Cell culture studies

Mouse neuroblastoma (Neuro2a) cells were obtained from ATCC (Rockville, MD) and cultured in DMEM containing 10% fetal bovine serum, penicillin (100 IU/ml) and streptomycin (100 μg/ml). To mimic the *in vitro* ischemia/reperfusion environment, Neuro2a cells were maintained in a hypoxic condition (94% N_2_, 5% CO_2_, 1% O_2_, 37 °C) in oxygen glucose deprivation media (OGD, Life Technologies) for 24 h. The cells were then re-oxygenated in DMEM supplemented with 10% FBS (5% CO_2_, 20% O_2_, 37 °C) for another 24 h.

To confirm co-localization of FBP and Fas, Neuro2a cells maintained in hypoxic conditions with oxygen glucose deprivation media for 24 h and then blocked with PBS containing 1% BSA and 0.05% Tween 20 for 2 h at 37 °C and incubated with anti-Fas antibody (ab82419, Abcam) and A_647_-conjugated FBP for 2 h at 4 °C. The cells were then washed and stained with a secondary FITC-conjugated anti-rabbit IgG antibody (ab97050, Abcam) and counterstained with Hoechst 33342. The cells were mounted in an aqueous mounting solution (Abcam, Cambridge, UK) and images obtained with a Leika TSP-SP5 confocal microscope. To analyze functional effects of FBP, normoxia and hypoxia-induced Neuro2a cells were treated with FBP at 1 mM for 4 h followed by exposure to FasL (10 nM). After 4 h, the cells were stained with Annexin V- PE (Apoptosis detection kit; BD Pharmingen^TM^) according to the manufacturer’s instruction.

Western blotting of Neuro2a cell lysates was performed with 50 μg of protein transferred onto nitrocellulose transfer membrane from a 12% SDS-PAGE. The blots were probed with rabbit antibodies to cleaved murine caspase-3 and caspase-8 (ab52293 and ab25901 respectively, Abcam) and a secondary polyclonal antibody to rabbit IgG coupled to HRP (ab97051: Abcam). The blots were developed using ECL western blotting substrate (Promega, Medison, WI).

### Animal studies

All experiments were performed in compliance with guidelines and using protocols approved by Institutional Animal Care and Use Committee (IACUC) of the Hanyang University. Acute cerebral ischemia was produced by a 1 h occlusion of the right middle cerebral artery (MCAO) in Sprague–Dawley rats weighing 280–320 g (Orient Bio, Seoul, Korea as previously described^66^. Briefly, ischemic stroke injury was induced by ligating the external carotid artery (ECA) with a silk thread followed by occlusion of middle cerebral artery (MCA) through the ECA to internal carotid artery (ICA) by inserting a nylon suture. Then, the common carotid artery (CCA) was completely occluded using a clip. After 1 h of occlusion, reperfusion was initiated by pulling out the suture. Regional cerebral blood flow (rCBF) was measured using a laser Doppler flowmetry instrument (Periflux System 5010, Jarfalla, Sweden) with a flexible probe affixed to the skull (2 mm posterior and 5 mm lateral to bregma) as described previously^67^. Intranasal administration of peptides at 12 h post-MCAO was performed using a pressurized olfactory device (POD; Impel Neuropharma, Washington, USA). Briefly, rats anesthetized with 5% isoflurane were placed in a supine position for dosing. The POD tip was carefully inserted into each nostril and then a catheter tube pre-filled with 25 μL volume of dose was slowly inserted for about 2 cm inside. The peptide solution was then sprayed using the POD.

For bio-distribution studies, A_488_-conjugated FBP was intranasally (IN) administered at 500 μg (1.4 mg/kg) in a final volume of 50 μL (25 μL in each nare) using the POD at 12 h post-MCAO and fluorescence analyzed at designated time points in isolated organs using Image Station 4000 mm (Carestream Rochester, NY) or by flow cytometry of single cell suspensions on a FACS Calibur™ (BD Bioscience, San Jose, CA) and analyzed using the Flowjo software.

In functional studies, rats subjected to MCAO were IN treated with 700 μg (2.0 mg/kg) of FBP in a 50 μL volume (25 μL in each nare). Neurological deficits were evaluated as described previously^36^. Briefly, rats that showed no apparent neurologic impairment were graded as 0. If the animals displayed forelimb flexion, they were awarded as grade 1. A grade 2 was given to animals that displayed a weak gripping power when pulled by their tails. A grade 3 was assigned to animals that circled to paretic side when pulled by tail when they were placed in enough space to move freely. If the animal spontaneously circled in free environment, a grade of 4 was assigned.

Brain pathology was analyzed by incubating 2 mm thick brain slices in 2% 2,3,5-triphenyltetrazolium chloride (TTC staining, Sigma) and measuring the infarct volume after fixation using Image J software as previously described^8^. Paraffin-embedded brain sections were also subjected to H&E staining and immunohistochemistry after with an antibody to rat Fas (ab82419, Abcam). Apoptosis in de-paraffinized, rehydrated brain sections was analyzed by TUNEL staining using the *In Situ* Cell Death Detection Kit (Roche, Germany) in accordance with the manufacturer’s instructions.

### Statistical Analysis

Experimental groups had sizes of N = 3 or N = 6 depending on the experiments as detailed in Figure legends. Statistical test for comparison were performed as follows; comparison between two groups at each time point- Mann-Whitney U Test; comparison between >2 groups at each time point- Kruskal-Wallis Test; comparison among two groups across all time points- Two-way ANOVA. Graphpad Prism 5 software was used for the analysis. P < *0*.*05* was considered statistically significant.

## Electronic supplementary material

Supplementary Information
