# Supplementary material for: Intranasal delivery of a Fas-blocking peptide attenuates Fas-mediated apoptosis in brain ischemia
Source: Sci Rep. 2018 Oct 9;8:15041. doi: 10.1038/s41598-018-33296-z (PMC6178348; doi:10.1038/s41598-018-33296-z)
Supplement: Supplementary file 1 — Supplementary Information [file 41598_2018_33296_MOESM1_ESM.pdf]

Supplementary information for: Intranasal delivery of a Fas-blocking peptide attenuates Fas-mediated apoptosis in brain ischemia

Irfan Ullah[1,2,#], Kunho Chung[1,2,#], Jungju Oh[1,#,], Jagadish Beloor[2], Sumin Bae[1], Sangah Clara Lee[2,3], Minhyung Lee[1], Priti Kumar[2,]* and Sang-Kyung Lee[1,]*

[1]Department of Bioengineering and Institute of Nanoscience and Technology, Hanyang University, Seoul, Korea, [2]Department of Internal Medicine, Section of Infectious Diseases, Yale University School of Medicine, New Haven, CT, USA,[3]Department of Behavioral and Social Sciences, Brown University, Providence, RI, USA.

*Correspondence should be addressed to P.K (priti.kumar@yale.edu) or S.K.L (sangkyunglee@hanyang.ac.kr)

Running title: Nose to brain delivery of a Fas-blocking peptide for reversing ischemia

[#] These authors contributed equally

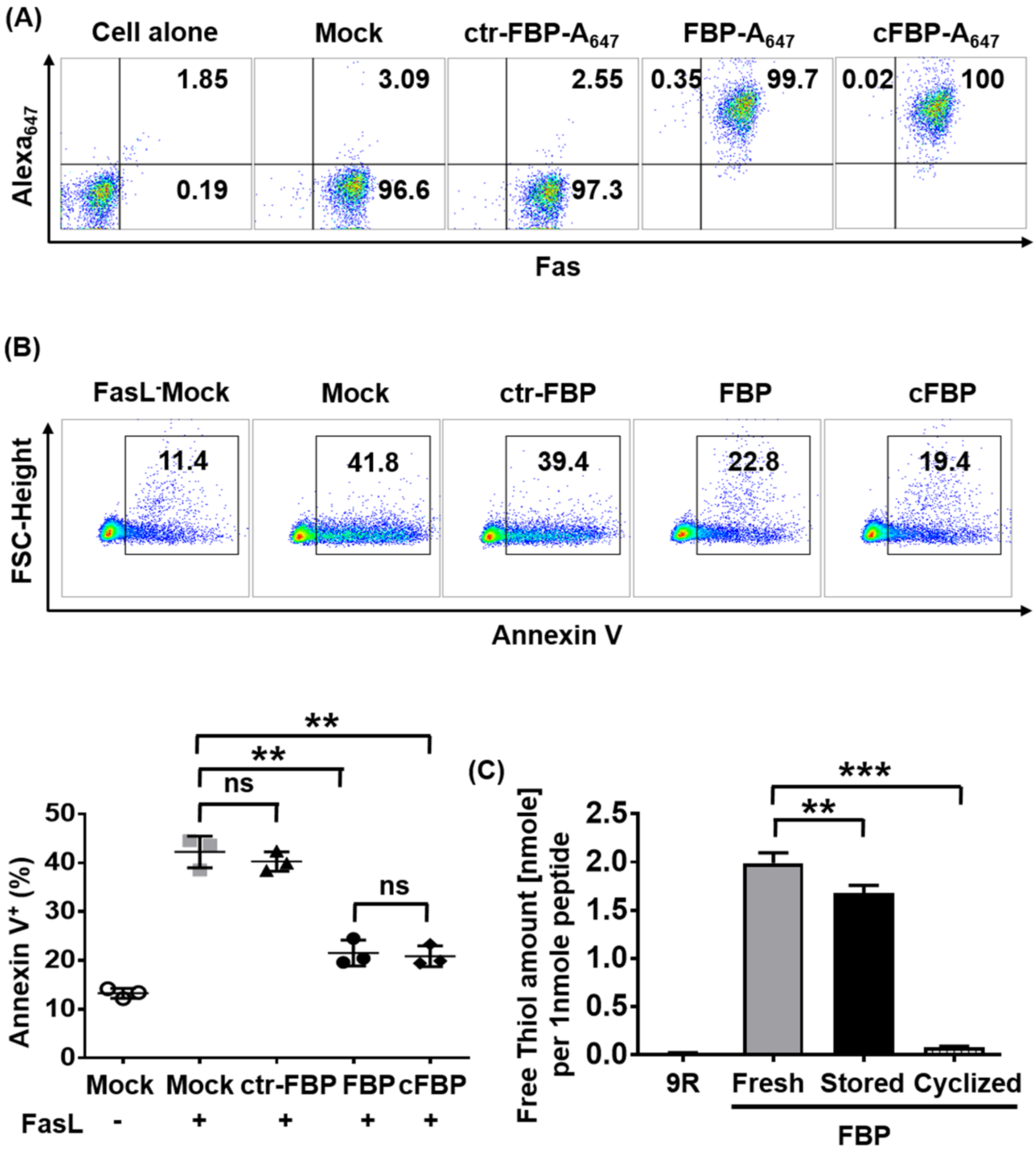

Supplementary Figure S1

(A) Flow cytometric analysis of Fas-expressing Jurkat cells bound to a rabbit anti-rat Fas antibody and the indicated A_{647}-peptides from three independent experiments. Cells untreated with Fas

antibody are indicated as cell alone and the cells stained anti-Fas antibody, A_{647}-labelled control peptide, A_{647}-labelled linear FBP peptide and A_{647}-labelled cyclic FBP peptide are indicated as Mock, ctr-FBP-A_{647}, FBP-A_{647} and cFBP-A_{647} respectively. (B) Anti-apoptotic effect of Fas-blocking peptide in Jurkat cells. Cells were exposed to FasL in the presence or absence of FBP. Representative dot plots are shown (upper panel) and scatter plots depicting % annexin V positive cells (lower panel). The data represent mean ± SD (N= 3 experiments). $^{**}P<0.01$, Mann-Whitney U test, $^{\#\#\#}P<0.001$; ns- not-significant. (C) Free thiol group analysis of 9R peptide (no free thiol), fresh (FBP freshly thawed), stored (FBP incubated for 10 days in PBS (pH 7.4) and cyclized (FBP incubated for 10 days in ammonium carbonite (pH 8.0). The data represent mean ± SD (N= 3 replicates). $^{**}P<0.01$ $^{***}P<0.001$; Mann-Whitney U test. FasL- Fas ligand, ctr-FBP- control peptide, FBP- linear Fas blocking peptide, cFBP- cyclic Fas blocking peptide.

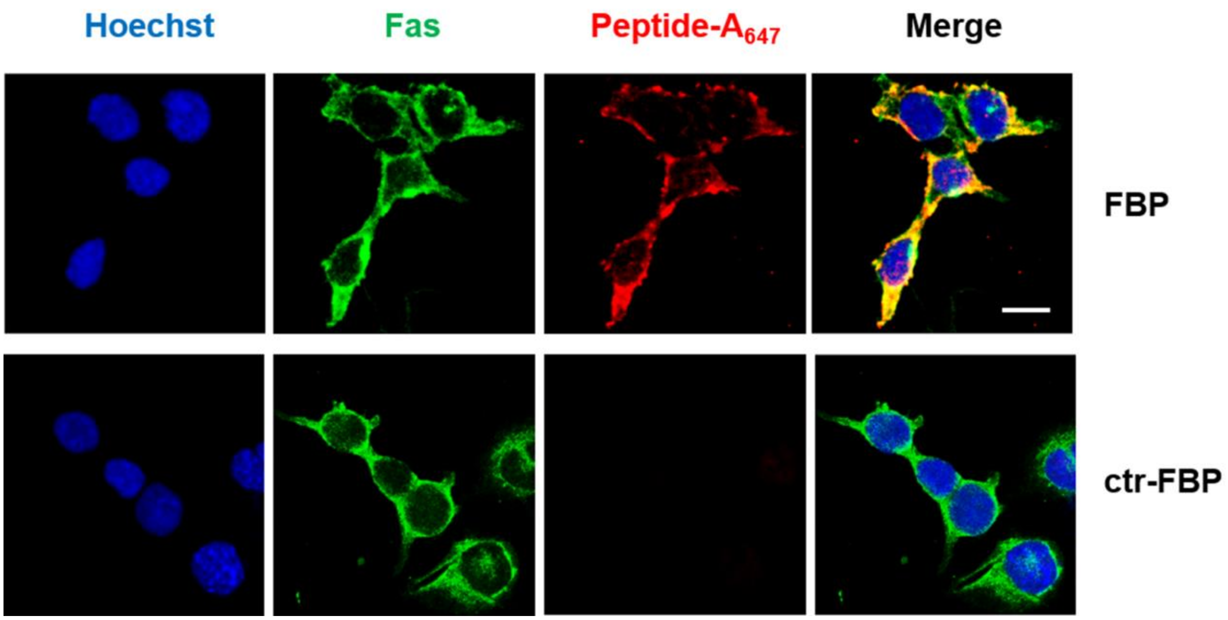

Supplementary Figure S2

Confocal images of hypoxic Neuro2a cells co-stained with A_{647}-labeled FBP or ctr-FBP (red) and a rabbit-antibody to mouse Fas (green) and Hoechst stain to detect nuclei (blue). Scale bar =10 µm

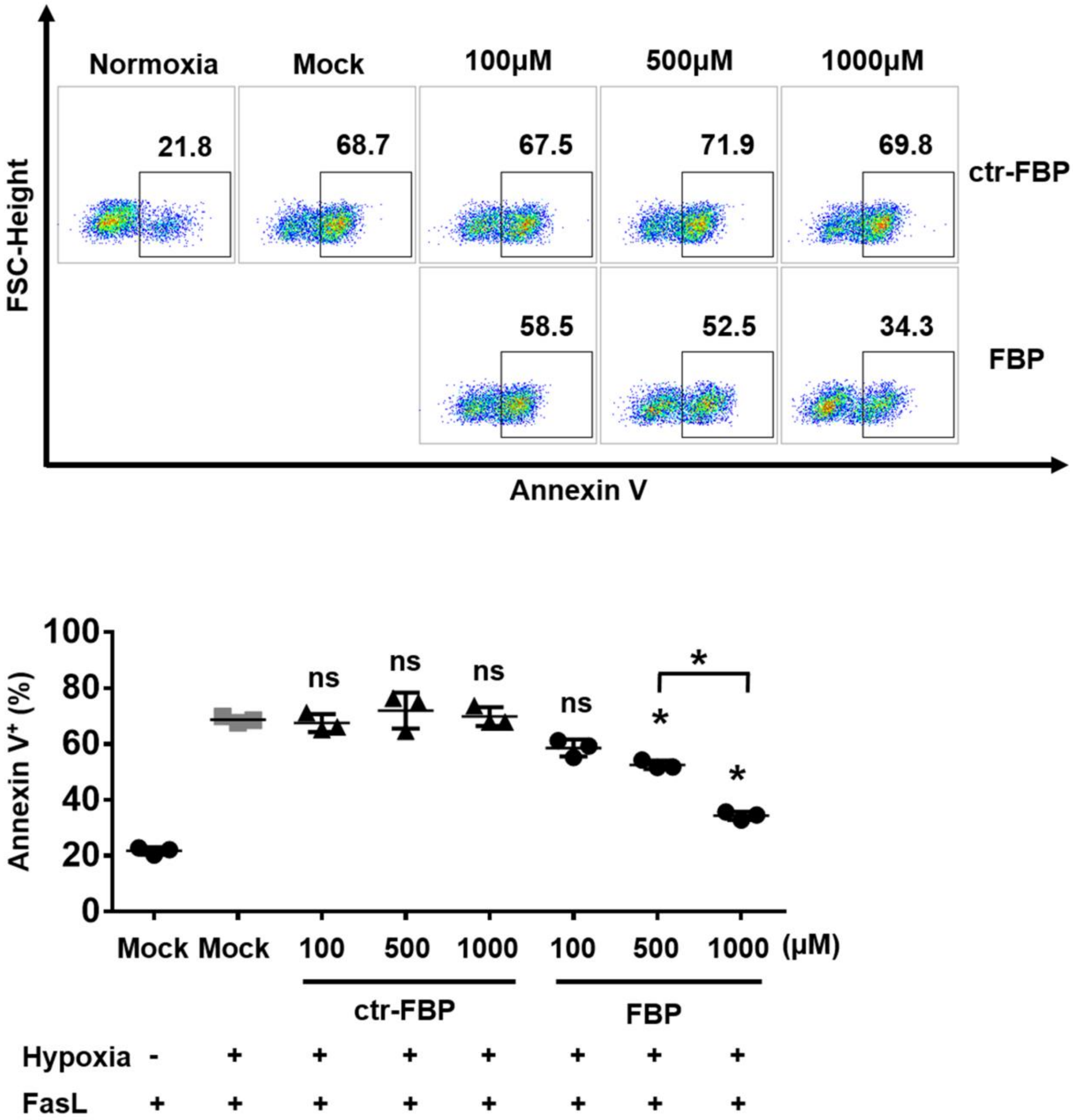

Supplementary Figure S3

Anti-apoptotic effect of Fas-blocking peptide in Neuro2a cells. Cells were exposed to FasL in the presence of increasing concentrations of FBP or a control peptide, ctr-FBP. Representative dot plots (upper panel) and scatter plots depicting % annexin V positive cells (lower panel). The data represent mean ± SD (N= 3 experiments). *$P<0.05$; Mann-Whitney U test, ns- not significant. FasL- Fas ligand, ctr-FBP- control peptide, FBP- linear Fas blocking peptide, cFBP- cyclic Fas blocking peptide. Normoxia represents Neuro2a cells maintained under normal culture conditions and hypoxia represents Neuro2a cells maintained for 24 h in OGD media under hypoxic condition followed by a 4 h re-oxygenation period.

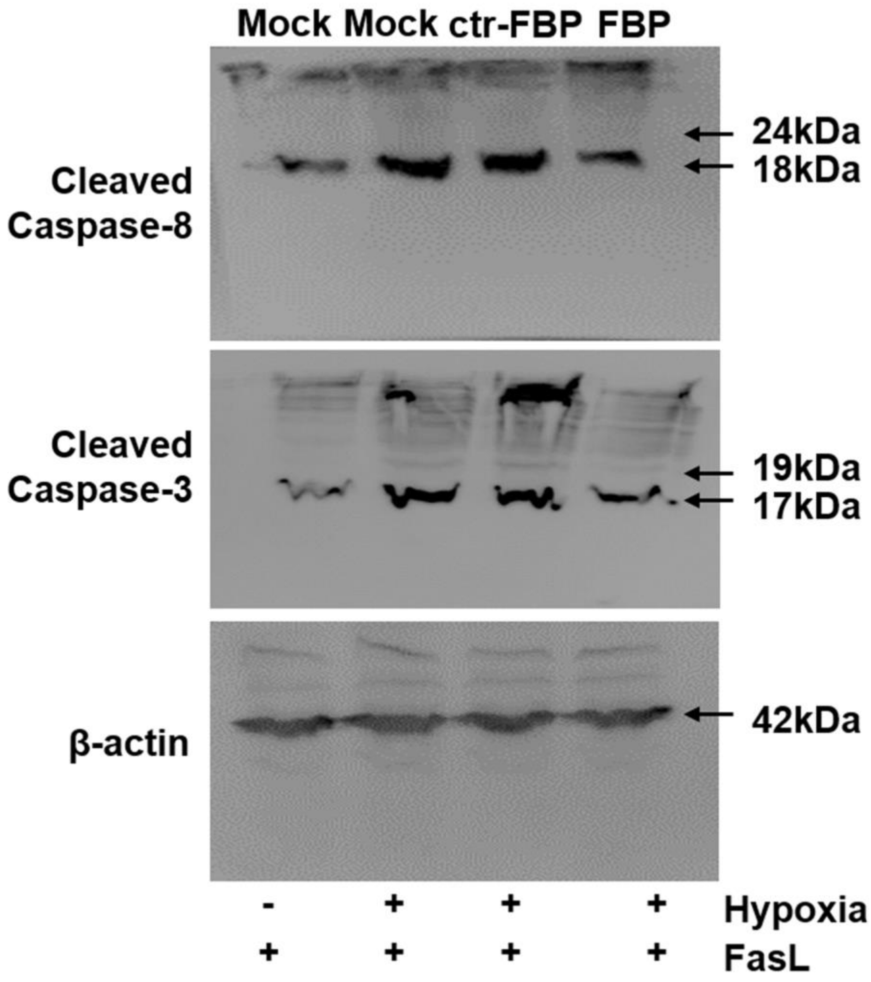

Supplementary Figure S4

Full scan blots from Figure 2C for cleaved effector caspase-3, caspase-8 proteins and β-actin.

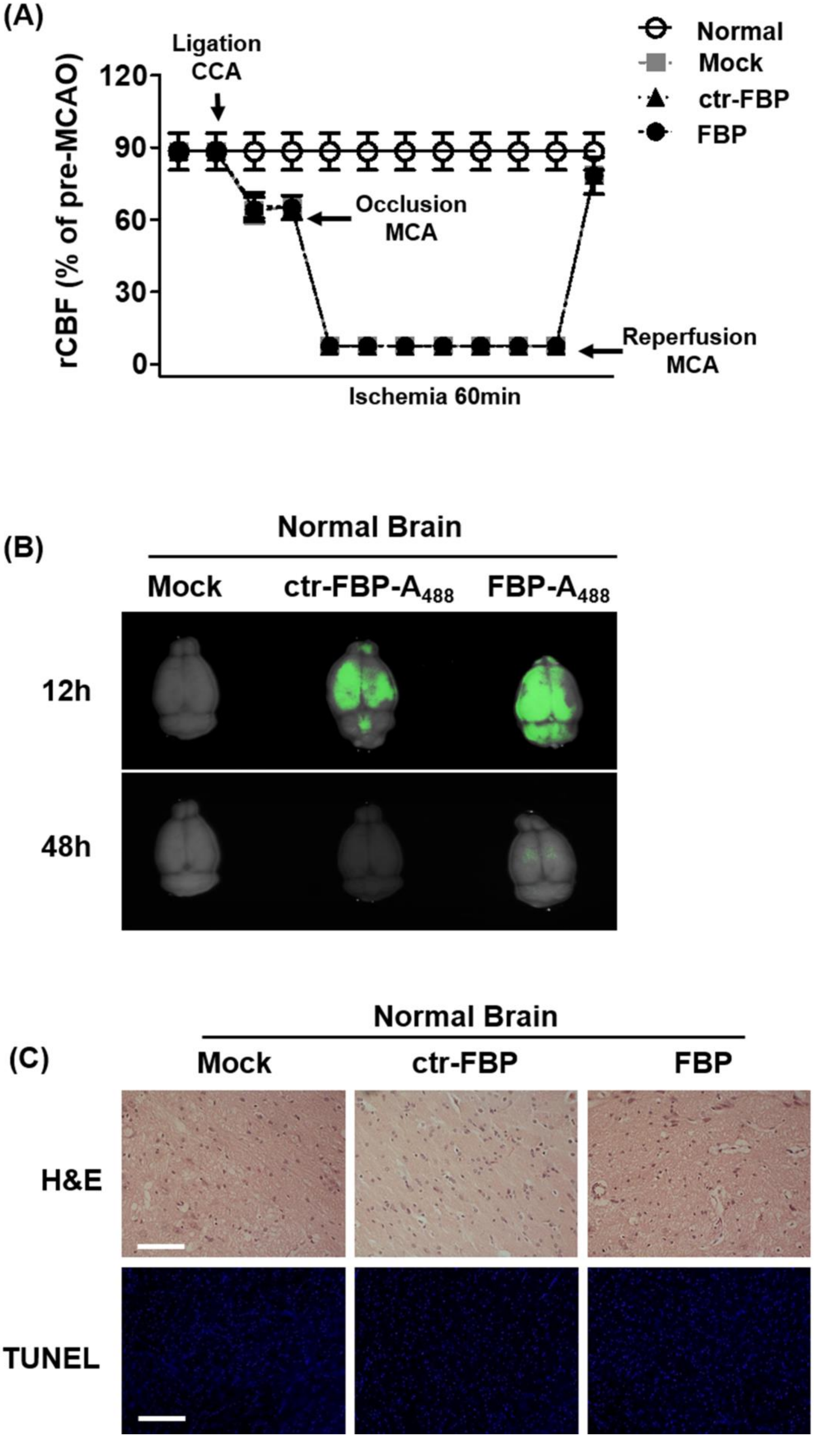

(A)
Ligation CCA
Occlusion MCA
Reperfusion MCA
rCBF (% of pre-MCAO)
120
90
60
30
0
Ischemia 60min
Normal
Mock
ctr-FBP
FBP
(B)
Normal Brain
Mock
ctr-FBP-A488
FBP-A488
12h
48h
(C)
Normal Brain
Mock
ctr-FBP
FBP
H&E
TUNEL

Supplementary Figure S5

(A) Regional cerebral blood flow (rCBF) in rat's subject to MCAO. The cerebral blood flow was measured during ligation of CCA, occlusion of MCA and after reperfusion (N=6 per group) and a 9 fold reduction was considered as a confirmation of ischemic stroke injury. (B) Bio-distribution of IN inoculated ctr-FBP-A_{488} or FBP-A_{488} at the indicated time points in normal non-ischemic rats that constitute a control group (N=3 per group). Mock treated animals were inoculated with PBS. (C) Representative hematoxylin and eosin (H&E) (upper panel) and TUNEL (lower panel) stained paraffin embedded sections from normal rat inoculated with PBS (Mock), ctr-FBP or FBP groups (N=6 per group). The tested normal rat with either ctr-FBP or FBP did not shows any change in tissue morphology and toxicity. Scale bare represents 100µm. ctr-FBP-A_{488}- $Alexa_{488}$ labeled control peptide, FBP-A_{488}- $Alexa_{488}$ labeled Fas-blocking peptide.
